# Comparative Evaluation of the Fracture Strength of Pulpotomized Primary Molars: An *In Vitro* Study

**DOI:** 10.5005/jp-journals-10005-1576

**Published:** 2019

**Authors:** Noorjahan Mohammad, Snigdha Pattanaik, Thimma BV Reddy, Dwitha Animireddy, Swetha Ankireddy

**Affiliations:** 1,5 Department of Pedodontics, Mamata Dental College, Khammam, Telangana, India; 2 Department of Orthodontics, Institute of Dental Sciences, Siksha O Anusandhan (Deemed to be University), Bhubaneswar, Odisha, India; 3,4 Department of Pedodontics, Panneya Dental College, Hyderabad, Telangana, India

**Keywords:** Calcium hydroxide, Cermet cements, Dental caries, Glass ionomer cements, Miracle mix

## Abstract

**Purpose:**

This study evaluates the fracture strength of pulpotomized primary molars restored with amalgam, miracle mix, cermet, resin-modified glass ionomer cement, and nanocomposites.

**Materials and methods:**

Fifty primary first and second molars were collected for this study. All the teeth were randomly divided into five groups (*n* = 10). Standard pulpotomy cavities were prepared. Teeth were air dried and the canal orifices were capped with a layer of zinc oxide eugenol. A lining of calcium hydroxide was placed over it. Amalgam, miracle mix, cermet, resin-modified glass ionomer cement, and nanocomposite were placed in groups I, II, III, IV, and V, respectively. All the samples were then subjected to the fracture strength test using the universal testing machine and the results were statistically analyzed.

**Results:**

All the groups were compared by the ANOVA one-way test which indicated that there were statistically significant differences among the five groups.

**Conclusion:**

Nanocomposites can be considered to be the best restorative material in terms of fracture strength among amalgam, miracle mix, cermet, and resin-modified glass ionomer cement.

**How to cite this article:**

Mohammad N, Pattanaik S, *et al.* Comparative Evaluation of the Fracture Strength of Pulpotomized Primary Molars: An *In Vitro* Study. Int J Clin Pediatr Dent 2019;12(1):5–9.

## INTRODUCTION

Humankind has always been plagued by the problem of restoring parts of the body lost due to disease or an accident. Dental caries is one of such prevalent chronic disease in children as well as in adults. Despite all the prevention strategies, childhood caries is still a fact that we confront every day. Carious primary teeth lead to pain inflammation and infection. The retention of pulpally involved primary teeth until the time of normal exfoliation always remains to be a challenge. Primary teeth with cariously exposed vital pulps should be treated with pulp therapies that allow for the normal exfoliation process.^1^ A pulpotomy is indicated in primary molars when the radicular pulp tissue is healthy or is capable of healing after surgical amputation of the affected or infected coronal pulp.^2^ Formocresol is the most commonly used pulp medicament, and it should be followed by immediate placement of a durable, stress-resistant restoration.

The restorative materials used after pulpotomy include amalgams, stainless steel crowns, intermediate restorative material (IRM), glass ionomer, and composite resin.^3^ Dental amalgam has been used for restoring teeth since the 1880s.^4^ For multisurface restorations in primary teeth, stain less steel crowns are superior to amalgams and have a success rate greater than that of amalgams in children under the age of 4 years.^5^

For the placement of stainless steel crowns, extensive tooth preparation with subgingival extension is required which can damage the surrounding periodontal tissues. A restoration which preserves the remaining tooth structure should utilize intracoronal retention which is critical to success and it helps in the survival of a damaged, pulpally treated, and valuable tooth. Amalgam lacks the desirable property of bonding to the tooth structure.^6^ Adhesive restorative materials improve the tooth resistance to fracture upon occlusal loading. The recent advances in adhesive technology and the introduction of stronger adhesive materials created conservative, highly aesthetic restorations that bond to the tooth structure and strengths it. The introduction of new bonding agents has also led to the possibility of restoring pulpotomized teeth with a bonded restoration instead of a crown. The ability to restore pulpotomized primary molars to their original strength and fracture resistance without the placement of crown could provide potential prosthesis, promote better marginal adaptation, and improve aesthetics.^7,8^

Pediatric dentistry requires adhesion of restorative materials to the tooth structure and it should be quickly placed. Wilson and Kent developed glass ionomer cements having aesthetic, adhesive, biocompatible, and anticariogenic properties. This material was developed by combining strength, rigidity, and fluoride release properties of a silicate glass powder with the biocompatibility and adhesive characteristics of a polyacrylic acid liquid.^9^

The addition of silver–amalgam alloy powder (miracle mix) to conventional materials increased the physical strength of the cement, resistant to abrasion, fracture resistant, and provided radiopacity.^10^ Silver particles were sintered onto the glass, and a number of products then appeared where the amalgam alloy content had been fixed at a level claimed to produce optimum mechanical properties for a glass cermet cement.^11^

Conventional glass ionomer cements have moisture sensitivity and lack of command cure. To overcome these problems, attempts have been made to combine glass ionomer chemistry with the well-known chemistry of composite resins. Resin modification of glass ionomer cement was designed to produce favorable physical properties similar to those of resin composites while maintaining the basic features of the conventional glass ionomer cement.^12^ The application of nanotechnology to composite resins has been one of the very important advances of the last few years in composite resin restorations. Nanotechnology is based on the production of functional materials and structures in the range of 100 nm using various physical and chemical methods and these nanocomposites having certain advantages such as reduced polymerization shrinkage increased mechanical properties and increased resistance to fracture.^13^

It was the objective of this study to evaluate the fracture resistance of pulpotomized primary molars restored with various restorative materials which can bear maximum occlusal loads, conserving the remaining tooth structure and bond to the tooth.

## MATERIALS AND METHODS

**Fig. 1:**
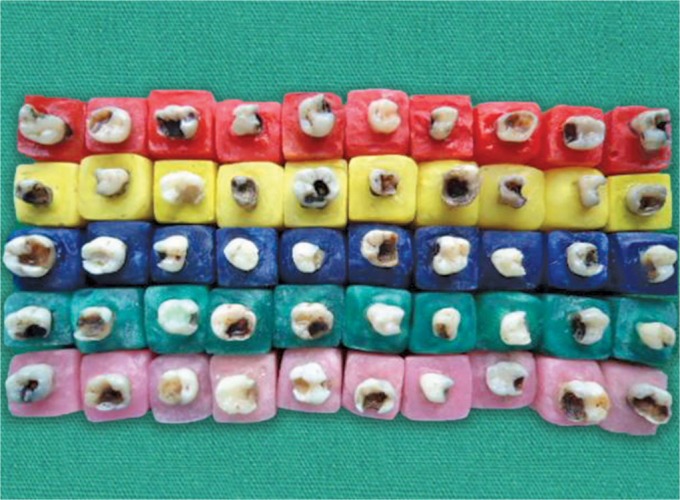
Samples mounted in acrylic

Fifty primary first and second molars indicated for the extraction due to caries were collected for this study. The collected teeth were stored in distilled water at the room temperature for not more than 3 months. All the teeth were randomly divided into five groups of 10 each. The samples were placed in the rectangular aluminum molds containing a thin mix of acrylic resin in such a way that the facial and the lingual cusps of the teeth were in the same plane. The acrylic resin was placed up to 1–2 mm of the tooth surface below the cement–enamel junction to approximate the height of healthy alveolar bone (Fig. 1).

**Fig. 2:**
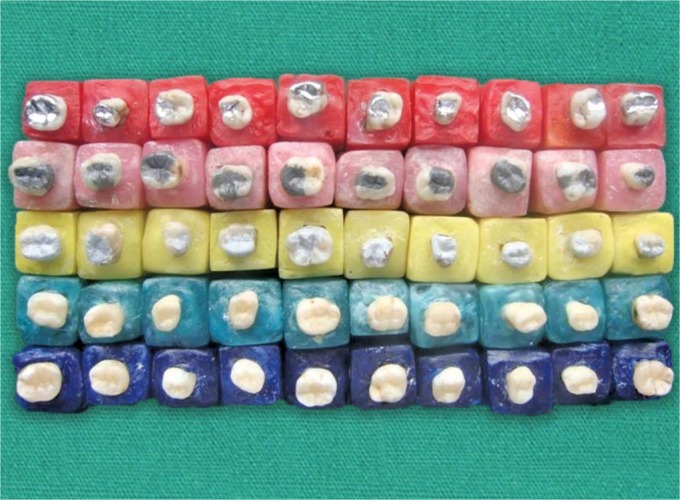
Completed specimens

Initially, caries was removed with a slow-speed round bur under a water coolant without entering the pulp chamber. The cavity size varied according to the extent of the decay. With the completion of the cavity outline, access to the pulp chamber was gained with the high-speed bur. A no. 6 carbide round bur in a slow-speed handpiece completed the final convenience form of the pulp chamber exposing the canal orifices, teeth were air dried, and the canal orifices were capped with a layer of zinc oxide eugenol. A lining of the fast setting Ca(OH)_2_ was placed over it to protect the remaining pulp from the irritants of restorative material and the walls were cleaned of any calcium hydroxide, using a sharp small excavator. The restorative materials were placed in the prepared cavity as follows (Fig. 2).

### Group I: Amalgam (Dental Products of India Ltd (DPI) Alloy)

Amalgam powder and liquid were mixed in a mechanical amalgamator to achieve a homogeneous consistency and the mixing time was 10 seconds. The triturated amalgam was then condensed into the prepared cavity after squeezing out of the excess mercury. Finally, carving was done to reproduce the proper tooth anatomy and then burnishing to smoothen the rough margins and surface of the restoration.

### Group II: Miracle Mix (GC)

About two to three scoops of powder and two drops of liquid were dispensed on a paper pad and mixing was done using a spatula. The working time was 1 minute and 30 seconds at 23°C. After achieving heavy consistency, it was condensed into the prepared cavity. The restoration was then carved to reproduce the proper tooth anatomy.

### Group III: Cermet (HI Dense—Shofu Dental Corporation (SHOFU))

Three scoops of powder and two drops of liquid were dispensed on a glass slab. Mixing was done by using a cement spatula. The mixing time was 30 seconds. After achieving a uniform and heavy consistency, then it was condensed into the prepared cavity. The restoration was then carved to reproduce the proper tooth anatomy.

### Group IV: Resin-modified Glass Ionomer Cement (GIC) (Vitremer)—3M ESPE

Two scoops of powder and two drops of liquid were dispensed and mixing was done by using a cement spatula. The mixing time was 45 seconds. The primer was applied for 30 seconds on the cavity, then it was air dried for 15 seconds and then it was cured for 20 seconds. The resin-modified GIC was placed above it and it was cured for 40 seconds. Polishing was done; after that, the finishing gloss was applied and cured for 20 seconds.

### Group V: Nanocomposites (Teric N-ceram)

The prepared pulpotomy cavity was treated with 37% phosphoric acid for 15 seconds, rinsed with water for 20 seconds, dried optimally to remove the excess water leaving a moist surface. The bonding agent was applied for 30 seconds and light cured for 20 seconds. Nanocomposite resin was placed in the prepared cavity and light cured for 40–60 seconds. Finishing and polishing were done.

**Fig. 3:**
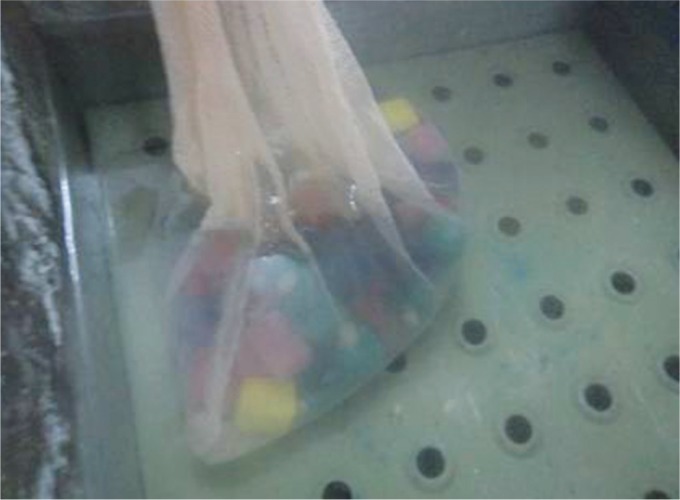
Thermocycling

**Fig. 4:**
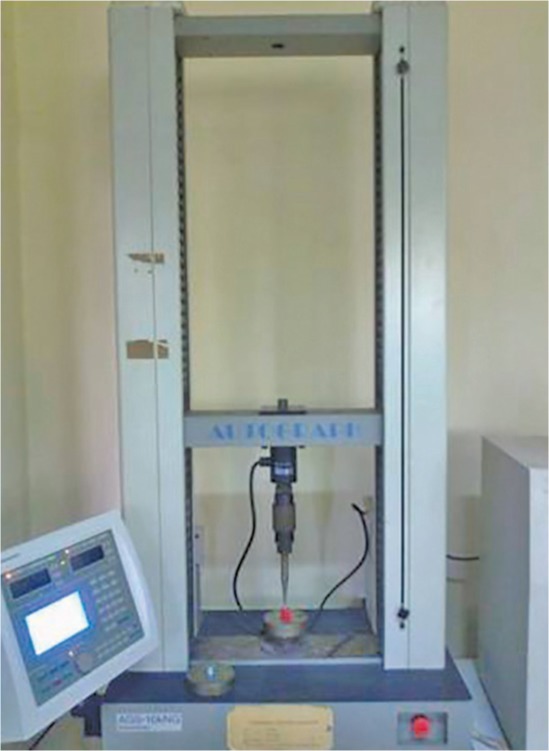
Measuring fracture strength by universal testing machine

After completion of all the five groups, the samples were stored in artificial saliva at the room temperature before being subjected to thermocycling (Fig. 3). The teeth were subjected to 1,000 thermocycles between 50 and 55°C with a dwell time of 30 seconds at each temperature. All the five groups were then subjected to the fracture strength test using a universal testing machine. Different sized tapered steel cones with a diameter of 3.5 mm for the primary first molars, 4.5 mm for the lower primary second molars, and 5.5 mm for the primary upper second molars were used. The teeth were tested to compression at a speed of 5.0 mm/minute and the breaking load was measured by recording the reading on the display panel of the machine (Fig. 4).

## RESULTS

**Fig. 5:**
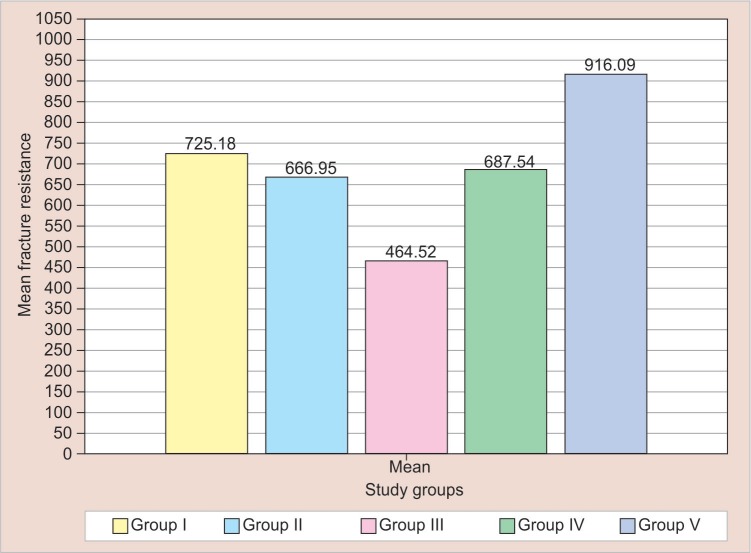
Comparison of means of fracture strength values

In the primary dentition when the mean fracture strengths of the amalgam, miracle mix, cermet, resin-modified glass ionomer cement, and nanocomposites were compared, nanocomposites showed the higher fracture strength which is significantly higher when compared with amalgam, miracle mix, cermet, and resin-modified glass ionomer cement (Table 1 and Fig. 5). The statistical analysis is explained in Table 2.

## DISCUSSION

The preservation of primary teeth until their time of exfoliation is required to maintain arch length, masticatory function, and esthetics.^14^ The selection of an ideal restorative material for the restoration of pulpotomized teeth is among the goals of dental materials’ research because these teeth are more susceptible to fracture due to the great loss of tooth structure. Fracture resistance of teeth depends on two main factors which are dimensions of the prepared cavity and the restorative material. Hence, the restorative material should have adequate strength and retention to protect the teeth against masticatory forces and preserve the remaining tooth structure. Amalgam-restored teeth have less stability than intact teeth, whereas composite-restored teeth have the stability equal or even greater than that of intact teeth. Based on a study by Hood et al. in 1999, amalgam can act like a wedge in between buccal and lingual cusps and increase the risk of fracture. In contrast, bonded composite restorations decrease the deflection of cusps under occlusal forces and by distribution and transfer of functional tensions at the tooth/bonding interface; they have the potential to reinforce weak tooth structure.^15^ Bonded restorations splint the cusps together and decrease cusp flexure, preventing their subsequent separation by fracture. In addition, placement of a considerable amount of adhesive restorative material in the pulp chamber may provide additional reinforcement by altering the fulcrum of cuspal flexing.^16^

The main reasons for the preference of GIC as a restorative material over amalgam in primary teeth are chemical adhesion to enamel and dentine, caries-inhibiting effect, superior esthetics, and its biocompatibility. Metal-reinforced GIC was more resistant to breaking than the traditional glass ionomer cement but less resistant than the light cured glass ionomer cement. Resin-modified glass ionomers have improved wear resistance compared to the original glass ionomers and are appropriate restorative materials for primary teeth.^12,17^

The better performance of resin modified glass ionomer cement (RMGIC) over amalgam is because of its adhesive property and probably by water sorption and expansion of the material during setting. In our study, the RMGIC mean fracture strength is nearer to amalgam and there is no statistically significant. Hence, RMGIC is a better material and can be used as an alternative for amalgam in primary molars.^18^

During the last few decades, the increasing demand for esthetic dentistry has led to the development of resin composite materials for direct restorations with improved physical and mechanical properties, esthetics, and durability. Nanocomposites are available as nanohybrid types, containing milled glass fillers and discrete nanoparticles, and as nanofill types, containing both nanosized filler particles, called nanomers, and agglomerations of these particles described as “nanoclusters.” The nanoclusters provide a distinct reinforcing mechanism compared with the conventional composites, significantly improving the strength and resistant to fracture.^19,20^

**Table 1:** Mean fracture strength and standard deviation in groups I–V

*Groups*	*No. of samples*	*Mean*	*SD*	*Min*	*Max*
Amalgam	10	725.18	158.45	506.25	1119.5
Miracle mix	10	666.95	173.62	410.5	884.25
Cermet	10	464.52	226.31	320	1096.85
RMGIC	10	687.54	175.09	423	1012
Nanocomposites	10	916.09	162.84	747.05	1357.2

**Table 2:** Analysis of variance (ANOVA) for fracture strength of five restorative materials used in the study

*Source of variation*	*Degree of freedom*	*Sum of squares*	*Mean sum of squares*	*Variance ration F value*	*p value*
Between groups	1,039,131	4	259782.9	7.67623	<0.0001 significant
Within groups	1,522,913	45	33842.5		
Total	2,562,044	49			

Statistical analysis: ANOVA one-way test

Statistically significant if *p* < 0.05

The mean value of maximum bite force has been reported between 151.9 and 374.4 N in different studies. In our study, the mean fracture resistance was 916.90, 725.18, 687.53, 666.95, and 464.52 N for nanocomposites, amalgam, resin-modified GIC, miracle mix, and cermet, respectively, which were much higher than the maximum bite force values reported in the literatures.^21–23^

## CONCLUSION

The materials used in dentistry for the restoration of primary and permanent teeth need to possess some properties such as adaptation to the cavity walls, a similar thermal expansion coefficient to that of teeth, biocompatibility, high fracture resistance, anti-cariogenic, and economical. Research is conducted in restorative materials to know the resistance to masticatory forces, resistance to abrasion, elasticity module, and contraction and tension stresses. The present *in vitro* study was done to compare and evaluate the fracture strength of amalgam, miracle mix, cermet, RMGIC and nanocomposites in pulpotomized primary molars. Nanocomposites have got the highest fracture strength followed by amalgam, RMGIC, miracle mix, and then cermet. Due to our statistical finding, RMGIC showed the fracture strength nearer to amalgam and it can be used in all restorations, particularly in primary teeth. Nanocomposites can be considered to be the best restorative material in terms of fracture strength among amalgam, miracle mix, cermet, and RMGIC.
